# Education and adolescent cognitive ability as predictors of dementia in a cohort of Danish men

**DOI:** 10.1371/journal.pone.0235781

**Published:** 2020-08-06

**Authors:** Else Foverskov, M. Maria Glymour, Erik Lykke Mortensen, Merete Osler, Gunhild Tidemann Okholm, Rikke Lund

**Affiliations:** 1 Department of Public Health, University of Copenhagen, Copenhagen, Denmark; 2 Center for Healthy Aging, University of Copenhagen, Copenhagen, Denmark; 3 Department of Epidemiology and Biostatistics, University of California San Francisco, San Francisco, California, United States of America; 4 Center for Clinical Research and Prevention, Bispebjerg and Frederiksberg Hospital, Copenhagen, Denmark; University of Oxford, UNITED KINGDOM

## Abstract

**Background:**

An association between education and dementia is well-established but it is unclear whether education is associated with dementia after accounting for early life cognitive ability and whether there is a joint effect, such that the risk associated with one of the exposures depends on the value of the other. We examined separate and joint associations of adolescent cognitive ability and educational attainment with risk of dementia among Danish men born between 1939 and 1959.

**Methods:**

Men (N = 477,421) from the Danish Conscription Database were followed for dementia from the age 60 for up to 17 years via patient and prescription registry linkages. Exposure measures included cognitive ability assessed at the conscript board examination around age 18 and highest educational level (low: 0–10 year, medium: 10–13 years, high: ≥13 years) at age 30 from registry records. Associations with dementia diagnosis were estimated in Cox proportional hazards models adjusted for birth year and age at conscript board examination. Interaction was assessed on the multiplicative scale by including a product term between the two exposure measures and on the additive scale by calculating relative excess risk due to interaction (RERI) between different levels of the exposure measures.

**Results:**

Compared to men in the high education group hazard ratio [HR] for men in the medium and low group were 1.21 (95% confidence interval [CI]: 1.13, 1.30) and 1.34 (95% CI: 1.24, 1.45), respectively when not adjusting for cognitive ability. Additional adjustment for cognitive ability attenuated the magnitude of the associations, but they remained significant (education medium: HR = 1.10, 95% CI: 1.02, 1.19 and education low: HR = 1.12, 95% CI: 1.02, 1.22). A 10% higher cognitive ability score was associated with a 3.8% lower hazard of dementia (HR = 0.962; 95% CI: 0.957, 0.967), and the magnitude of the association only changed marginally after adjustment for education. Men in the low education group with relatively low cognitive ability were identified as a high-risk subgroup for dementia. The increased risk associated with exposure to both risk factors did, however, not significantly depart from the sum of risk experienced by men only exposed to one of the risk factors (estimates of RERI were not significantly different from 0) and no significant evidence of either additive or multiplicative interactions was found.

**Conclusions:**

In conclusion, the results suggest that education and cognitive ability protect against the risk of dementia independently of one another and that increases in educational attainment may at least partially offset dementia risk due to low cognitive ability.

## Introduction

Dementia is a major population health concern and is globally estimated by The World Health Organization to affect 5–8% of the general population aged 60 and above [1]. Dementia is a syndrome defined by considerable deterioration in cognitive functioning commonly leading to social and physical disability and dependency. The immense cost of dementia for individuals directly affected, their families and society at large call for preventive strategies focusing on modifiable risk factors and high-risk subgroups in the population.

Risk of dementia in old age is elevated among people with a lower level of educational attainment [2, 3]. Education may influence occurrence of dementia though behavioral patterns affecting vascular health or through level of cognitive reserve, which is hypothesized to affect people’s ability to cope with neurodegenerative pathology [4, 5]. Lower levels of early life cognitive ability, although there are fewer studies, has also been found to be associated with a higher risk of dementia in old age [6–9] and the pathways linking cognitive ability and dementia may be similar to those linking education and dementia [4, 10]. Cognitive ability and education are closely interrelated and there is probably reciprocal causality across the life course [11]. However, neither of the two are a perfect proxy for the other. Factors like teaching quality, motivation, effort, personality traits and parental support are also likely to have substantial effects on educational attainment [12]. Other environmental factors influencing cognitive development may include maternal substance use during pregnancy, breastfeeding and early language exposure [13–15]. It is therefore relevant to study cognitive ability and education mutually to get a better understanding of both their separate and joint associations with dementia risk.

Currently few data sources with birth cohorts at risk of dementia in later life have access to information on both early life cognitive ability and education. Knowledge of whether a low educational level is a risk factor for dementia after accounting for early life cognitive ability is therefore limited and it is unclear if only cognitive or also non-cognitive educational related skills are relevant to dementia risk. Additionally, we know little about whether there is a joint effect of the two risk factors. That is, if the risk associated with one of the exposures depends on the value of the other. The strength of the association between early life cognitive ability and dementia risk may for example be conditional on education, with stronger associations seen for people with low compared to a high educational level. This is possible not only because continued education may lead to improved cognitive ability [16] but also because continued education may lead to improvements in other capacities of the individual such as personal control and agency [17] that may counteract the increased risk associated with relatively low early life cognitive ability.

In the present study we examine separate and joint associations of cognitive ability at age 18 and highest attained education at age 30 with risk of dementia from age 60 years in a cohort of Danish men followed for a maximum of 17 years.

## Material and methods

### Participants

The Danish Conscription Database (DCD), a cohort of 728,160 men born between 1939 and 1959, has been described in detail previously [18]. The database holds information from the compulsory conscript board examination that all Danish men must attend at around 18 years of age to be assessed for military service. We use a subcohort of 519,085 men who were 1) born between 1939 and 1956 and thus turned 60 before the end of follow-up on April 1st. 2016 (111,296 men excluded) and 2) did not die or migrate before turning 60 (77,030 and 20,749 men excluded respectively). Follow up for a dementia diagnosis was started at age 60 because the validity of dementia diagnosis from hospital registers among younger individuals has been found to be low [19]. Using the Danish personal identification numbers the DCD data was linked to registry data from Statistics Denmark and the Danish Health Data Authority. Data linkages and analysis were approved by the Danish Data Protection Agency.

**Fig 1:**
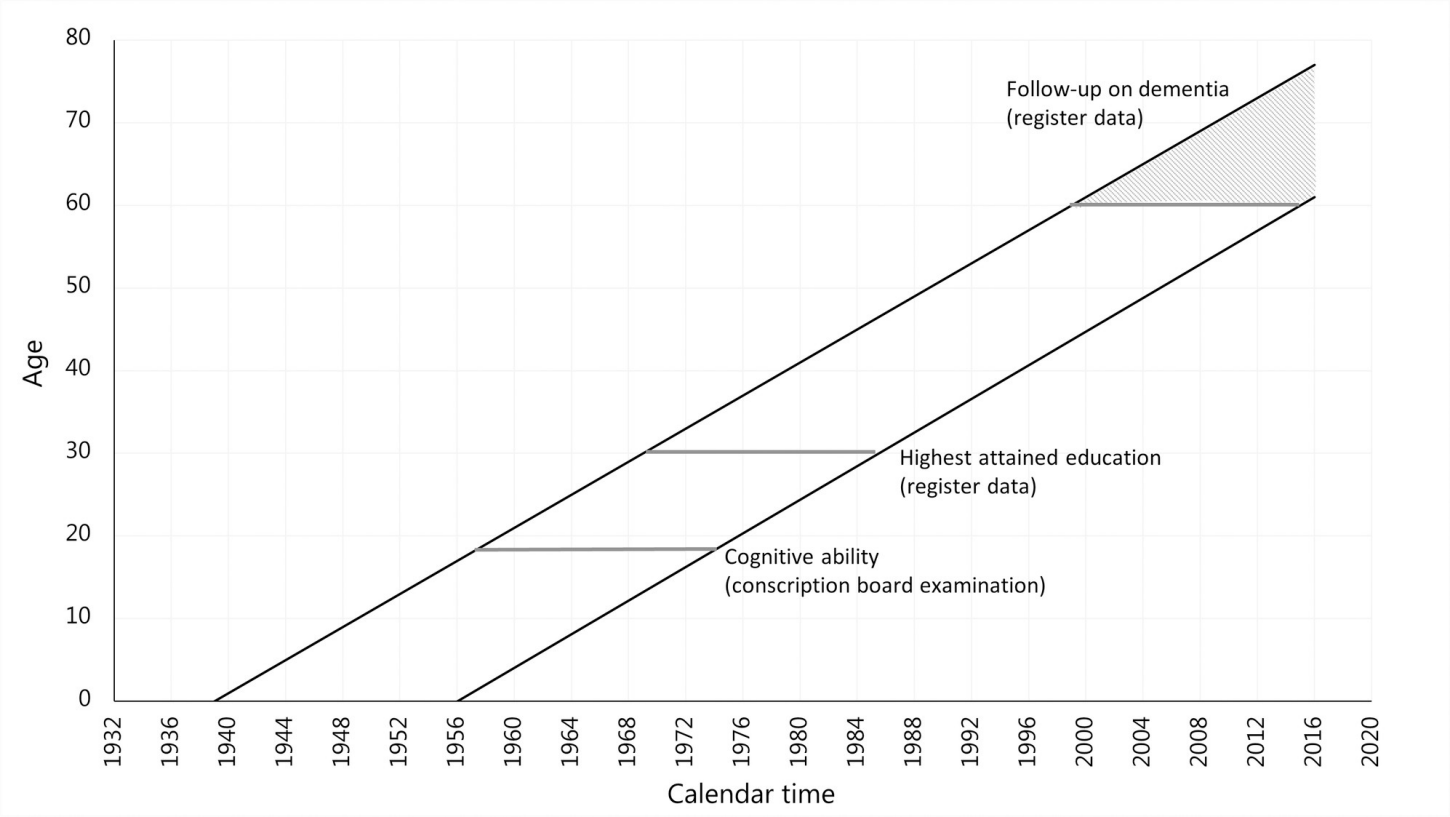
Data presentation by age and calendar time, sub-cohort of men from The Danish Conscription Database.

Of the 519,085 men in the sub-cohort 41,643 (8%) were excluded from the analysis. The largest proportion of cases excluded had missing conscript examination data (n = 28,151). This was mainly due to men being exempted from the examination if they volunteered for military service before their 18th birthday or had a medical condition that made them unfit for military service (e.g. intellectual disability, psychiatric disorders or epilepsy) [16]. The additional excluded cases were due to missing information on the time of the conscript board examination (n = 261), born in adjoining birth years before 1939 (n = 104), registered age less than 17 years at the time of the examination (n = 24), missing information on the cognitive test (n = 3,361), missing register information on educational attainment (n = 6,951) and registered with a dementia diagnosis before the age of 60 (n = 2791). This resulted in an analytical sample of 477,442 men. Fig 1 presents the study data.

### Cognitive ability

Cognitive ability was assessed at the conscript board examination with the Børge Prien Prøve (BPP). The test has previously been described in detail [20]. BPP is a group-administrated intelligence test in paper-and-pencil format lasting 45 minutes and comprising of four subtests: letter matrices (19 items), verbal analogies (24 items), number series (17 items) and geometric figures (18 items). The total test score (range 0–78) has been found to correlate highly with the Wechsler Adult Intelligence Scale [21].

### Education

Information on highest attained education through age 30 came from the Danish Educational Registry, which contains information from student registers from 1974 onwards combined with self-reported information for individuals completing their highest attained education before 1974 from the 1970 Population and Housing Census [22]. In the analytical sample the educational data of 63% was from the 1970 Population and Housing Census (i.e., 63% of the sample completed no additional education before age 30 beyond the level self-reported in the 1970 Census). Educational data from the registry was categorized as low (lower secondary; 0–10 years), medium (upper secondary general or vocational; 10–13 years), or high (short-, medium- and long-term higher education; ≥ 13 years).

### Dementia

The Danish Psychiatric Central Registry and the Danish National Patient Registry hold information from 1969 and 1977, respectively, on diagnosis and date for all hospital encounters including both inpatients, outpatients and emergency department contacts (until 1995 only inpatients, but thereafter also outpatients and emergency department contacts) [23, 24]. Diagnoses are classified according to the 8^th^ revision of the International Classification of Disease (ICD-8) until 1993 and from then on according to the 10^th^ revision (ICD-10). Men with a main diagnosis of dementia (ICD8: 290.00–290.99 and ICD10: F00.0-F03.9; G30.0-G30.9; G31.8; G31.9) were identified as dementia cases. The Danish National Prescription Registry was additionally used to identify dementia cases. The registry has been running since 1995 and includes data on dispensing date and Anatomical Therapeutic Chemical (ATC) codes for prescription drugs bought in pharmacies and drugs prescribed to nursing home residents [25]. Men who had redeemed prescriptions of anti-dementia drugs registered with ATC code N06D were identified as dementia cases (drug names included are memantine, donepezil, rivastigmine and galantamine).

Follow-up for dementia in the analytical sample started in the year 1999 where the first men in the sample turned 60 years of age, but data going back to the start of the different registers were used to identify and exclude men who had received a dementia diagnosis before age 60. The validity of dementia diagnosis obtained from the Danish Psychiatric Central Registry and the Danish National Patient Registry using ICD-10 in the elderly population has been found to be high [26]. The validity of dementia diagnosis based on ATC codes has not been examined. Among the 6,528 dementia cases registered in the analytical sample 3,731 were identified with ICD-10 codes, 508 with ATC codes and 2,289 with both ICD-10 and ATC codes. For the latter group the date of dementia diagnosis was set to the earliest date in the registers.

### Statistical analysis

First descriptive characteristics of study participants were assessed, both in the total sample and split by dementia status. Next, the associations between cognitive ability, education and risk of dementia were analyzed using Cox proportional hazards regression models with age as the underlying time scale. Entry time was the date the men turned 60 years of age and follow-up ended at the date of diagnosis of dementia, death, emigration or April 1^st^ 2016, whichever came first. Models were adjusted for birth year and age at conscript board examination to account for potential cohort effects and effects of increasing age at conscript board examination (potentially linked to increasing education) on cognitive test performance. We accounted for the effect of these two variables by stratifying on them in the Cox models, thus allowing baseline hazards to differ for different birth years and ages at the conscript board examination. Instead of assuming linearity in the log hazard of the cognitive ability score, fractional polynomials of degrees one and two were evaluated to find an appropriate functional form [27]. To allow for logarithms, the score was rescaled from range 0–78 to 1–79. The best fitting model was a one-term model with power 0, that is a model in which the natural logarithm of the cognitive ability score was used to predict dementia onset. The natural logarithm of the cognitive ability score was consequently used in the analysis meaning that the coefficients for the variable refers to a relative change in the outcome. The proportional hazard assumption was not found to be violated based on graphical examination of log-log plots and plots of Schoenfeld residuals. Associations between cognitive ability, education and risk of dementia were examined both separately (Model 1 and 2) and in a mutually adjusted model (Model 3). Interaction on the multiplicative scale was assessed by including product terms between the educational groups and cognitive ability in the mutual adjusted model (Model 4) and evaluated using a likelihood ratio test. Results from the latter model was additionally used to evaluate additive interaction measured as relative excess risk due to interaction (RERI) [28]. The specification of RERI used is in accordance with Rothman’s definition (RERI = HR_11_—HR10—HR_01_ + HR_00_ for two binary exposure variables) and the null hypothesis of RERI = 0 equals a test of whether the hazard ratio (HR) of experiencing both exposures is the same as the sum of the HR of experiencing one exposure alone [28, 29]. Having a continuous exposure variable, we calculated RERI between different levels of cognitive ability ranging from the 5^th^ to the 95^th^ percentile in the total population. Calculations were limited to values between the 5^th^ and 95^th^ percentile to ensure a sufficient number of cases in all educational groups. Stata version 16 was used for all statistical analyses and all data were anonymized prior to access and analysis. Tests for RERI were calculated using the nlcom command with standard errors obtained using the delta method.

## Results

The study cohort of 477,442 men were followed for an average of 7.7 (range 0–17) years from a baseline age of 60. During follow-up, 6,528 (1.4%) men were diagnosed with dementia. Table 1 shows descriptive characteristics of cognitive ability and educational attainment in the total study cohort as well as characteristics by dementia outcome. Dementia cases had a lower average cognitive ability score compared to the total population within each educational group and higher proportions of dementia cases were seen among men with low educational attainment compared to high. The coefficient of Spearman’s rank correlation between cognitive ability and education was -0.52 suggesting a fairly strong relationship.

**Table 1:** Characteristics of study participants.

	All^a^	Dementia cases^b^	
		Yes	No
Total, n (%)	477,442 (100.0)	6,528 (1.37)	470,914 (98.63)
Cognitive ability^c^, mean (SD)	37.80 (12.08)	34.98 (12.77)	37.84 (12.06)
Cognitive ability^c^ by education, mean (SD)			
High	48.38 (8.86)	47.69 (9.22)	48.39 (8.86)
Medium	37.49 (10.18)	35.30 (10.55)	37.52 (10.17)
Low	30.04 (11.33)	27.40 (11.58)	30.09 (11.32)
Education, n (%)			
High	103,537 (21.69)	1,216 (1.17)	102,321 (98.83)
Medium	242,484 (50.79)	3,146 (1.30)	239,338 (98.70)
Low	131,421 (27.53)	2,166 (1.65)	129,255 (98.35)
Birth year, n (%)			
1939–1943	116,517 (24.40)	3,647 (3.13)	112,870 (96.87)
1944–1947	132,450 (27.74)	1,929 (1.46)	130,521 (98.54)
1948–1951	115,110 (24.11)	747 (0.65)	114,363 (99.35)
1952–1956	113,365 (23.74)	205 (0.18)	113,160 (99.82)
Age at conscript examination, n (%)			
17–18	206,573 (43.27)	3,484 (1.69)	203,089 (98.31)
19–20	197,080 (41.28)	2,183 (1.11)	194,897 (98.89)
21 or older	73,789 (15.46)	861 (1.17)	72,928 (98.83)

^a^ Percentage sum to a hundred across columns.

^b^ Percentage sum to a hundred across rows.

^c^ Total test score from Børge Prien Prøve, range 0–78.

Estimated HR and 95% CI’s for cognitive ability and education predicting the incidence of dementia are shown in Table 2. To interpret the association between the natural log-transformed cognitive ability score and the hazard of dementia we calculated the HR associated with a 10% higher cognitive ability score equal to ln(1.10). This was done by multiplying the regression coefficient from Model 1, that is ln(0.67), with ln(1.10) and then exponentiating this number to get the hazard ratio. Thus, the HR of a 10% higher cognitive ability scores equaled exp(ln(0.67)*ln(1.1)) = 0.962. Lower and upper bounds of the confidence interval were calculated in the same way and equaled 0.957 and 0.967 respectively. As higher values are more favorable, a 10% higher cognitive ability score was associated with a 3.80% lower hazard of dementia when not adjusting for education (Model 1). Compared to men in the high education group the dementia hazard was significantly elevated for men in the medium (HR = 1.21, 95% CI: 1.13, 1.30) and low education group (HR = 1.34, 95% CI: 1.24, 1.45) when not adjusting for cognitive ability (Model 2). In the mutually adjusted model (Model 3) the estimates for cognitive ability only changed slightly (HR 10% change = 0.965, 95% CI: 0.959, 0.971). The estimates for education were considerably attenuated compared to Model 2 without adjustment for cognitive ability, but still significantly associated with hazard of dementia (education medium: HR = 1.10, 95% CI: 1.02, 1.19; education low: HR = 1.12, 95% CI: 1.02, 1.22).

**Table 2:** Associations between cognitive ability, education and hazard of dementia^a^.

	Model 1	Model 2	Model 3	Model 4^b^
	HR (95% CI)	HR (95% CI)	HR (95% CI)	HR (95% CI)
Cognitive ability (cog)				
Natural log transformed	0.67 (0.63, 0.71)		0.69 (0.64, 0.73)	0.75 (0.58, 0.96)
Education (educ)				
High		1.00	1.00	1.00
Medium		1.21 (1.13, 1.30)	1.10 (1.02, 1.19)	1.09 (1.01, 1.18)
Low		1.34 (1.24, 1.45)	1.12 (1.02, 1.22)	1.11 (1.01, 1.22)
Interaction terms				
Medium educ*ln(cog)				0.90 (0.69, 1.18)
Low educ*ln(cog)				0.91 (0.74, 1.19)

Abbreviations: CI = confidence interval; HR = hazard ratio.

^a^ Results from Cox proportional hazards models. N = 477,442 and dementia cases = 6,528. Model 1 adjusted for cognitive ability; Model 2 adjusted for education; Model 3 adjusted for both cognitive ability and education; Model 4 adjusted for both cognitive ability and education and including an interaction term between the two variables. All four models were additionally adjusted for categorical measures of birth year and age at conscript board examination (see categories in Table 1).

^b^ The natural log transformed cognitive ability score was centered at the 75th percentile equal to 3.87, and the main effects of education therefore shows associations for men with this cognitive ability score.

Interaction terms were included in Model 4 and to allow for a meaningful interpretation of the main effects for education the natural log transformed cognitive ability score was centered at 3.87 corresponding to the mean cognitive score among the highly educated men and the 75th percentile in the total cohort. No interaction was observed between cognitive ability and education on a multiplicative scale (Model 4, likelihood ratio test *P* value = 0.87).

**Fig 2:**
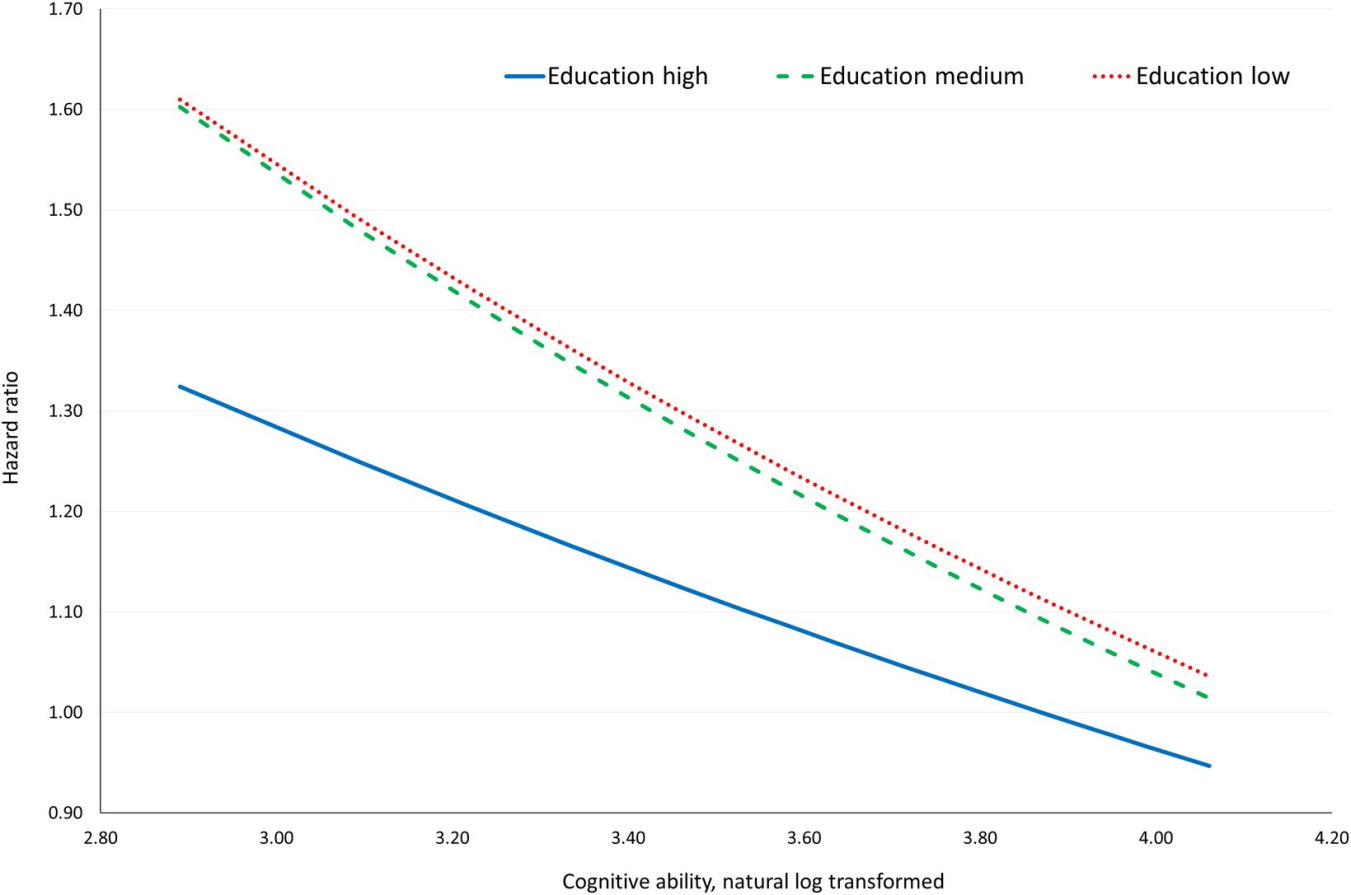
Predicted hazard ratios for dementia by educational level and cognitive ability. ^a^ Predicted hazard ratios calculated using estimates from Model 4 with education high and ln cognitive ability score of 3.87 as referent and presented for the three education groups at a range of cognitive ability scores covering the 5th to 95th percentiles. N = 477,442 and dementia cases = 6,528.

Fig 2 gives a graphical illustration of the predicted joint effects from Model 4 for each education group at a range of the cognitive ability score covering the 5^th^ to the 95^th^ percentiles in the total population. The results indicated that the difference in hazards between high and low educated men as well as between high and medium educated men decreased with increasing levels of cognitive ability. However, the curves in Fig 2 were estimated with considerable uncertainty, which can be seen from Table 3 where selected HR for predicted joint effects are presented along with 95% CI’s, and no significant additive interactions were found (RERI presented in Table 3). For example, comparing men with a low education and a cognitive ability score equal to the 5th percentile to men with a high education and a cognitive ability score equal to the 75th percentile an HR of 1.61 was estimated (95% CI: 1.47, 1.75) along with a positive but non-significant relative excess risk due to interaction of 0.17 (95% CI: -0.17, 0.51).

**Table 3:** Predicted hazard ratios for dementia by educational level and cognitive ability and tests for additive interactions (RERI)^a^.

Cognitive ability, ln transformed (percentile)^b^	Education high	Education medium		Education low	
	HR (95% CI)	HR (95% CI)	RERI (95% CI)	HR (95% CI)	RERI (95% CI)
2.89 (5)	1.32 (1.00, 1.65)	1.60 (1.44, 1.77)	0.19 (-0.17, 0.54)	1.61 (1.47, 1.75)	0.17 (-0.17, 0.51)
3.40 (25)	1.14 (1.01, 1.28)	1.31 (1.21, 1.41)	0.08 (-0.07, 0.22)	1.33 (1.22, 1.43)	0.07 (-0.07, 0.21)
3.66 (50)	1.06 (1.01, 1.12)	1.18 (1.10, 1.27)	0.03 (-0.03, 0.09)	1.20 (1.10, 1.31)	0.03 (-0.03, 0.09)
3.87 (75)	Referent	1.09 (1.01, 1.18)	-	1.11 (1.01, 1.22)	-
4.06 (95)	0.95 (0.90, 0.99)	1.01 (0.92, 1.11)	-0.02 (-0.07, 0.02)	1.04 (0.93, 1.14)	-0.02 (-0.07, 0.02)

^a^ Predicted hazard ratios calculated using estimates from Model 4 (see Table 2) with education high and ln cognitive ability score of 3.87 (75th percentile) as referent. N = 477,442, dementia cases = 6,528.

^b^ Percentile values apply to the total population.

## Discussion

In this study of 477,442 Danish men followed for a maximum of 17 years from aged 60, we confirmed that both lower levels of early life cognitive ability and educational attainment are associated with an increased dementia risk, and further found that the increased risk remained when the two risk factors were examined simultaneously. Men with relatively low cognitive ability in the lowest educational group had a higher risk of dementia compared to men with only one of the two risk factors, i.e., either low cognitive ability or low education. However, the combined exposure of both risk factors was not found to deviate significantly from the sum of risks in men exposed to one of the risk factors alone.

An association between adolescent cognitive ability and the risk of dementia from age 60 has previously been found using DCD data [6]. This prior research did not address the effect of education independently or in combination with a cognitive score. An additional new result of this study comes from using fractional polynomials to find an appropriate functional form for the cognitive ability score. After transforming the cognitive ability score using the natural logarithm, we found that a 10% increase in the score was associated with 3.80% decrease in the hazard of dementia. Because the HR of a fixed percentage increase does not depend on the baseline cognitive ability score the result implies that increases in cognitive ability among people in the lower end of the distribution may have the biggest preventive potential. This is due to the smaller absolute increases needed to obtain a 10% higher cognitive score in the lower compared to the higher end of the distribution. Cognitive ability measured at age 11 was similarly associated with dementia in later life in a case-control study of participants in the Aberdeen City 1932 Scottish Mental Survey [9], but two other studies using data from the 1921 Lothian Birth Cohort and the Helsinki Birth Cohort Study found no association between early life cognition and dementia [30, 31].

While numerous other studies have found education to be a risk factor for dementia [2], the results from this study are rare in showing that low education remains associated with an increased risk of dementia when accounting for cognitive ability in adolescence. Two related Swedish studies using school grades around 10 years of age as a proxy for cognitive ability reported that having a high or medium educational level compared to a low was associated with reduced risk of dementia among both men and women after adjustment of mean school grades [32, 33]. The protective effect was, however, not found to be significant for all comparisons, which may have been due to the relatively small sample sizes studied.

The finding of an association between education and dementia independent of cognitive ability suggests that not only cognitive aspects of education are important for dementia risk, but also non-cognitive aspects, which may be related to the influence of educational achievements on shaping healthy behaviors [34, 35], possibly by increasing effective agency and a sense of personal control [17]. Many observational cohort studies have reported a link between behavioral patterns and dementia and results from multidomain lifestyle interventions targeting people at an increased risk of dementia have shown beneficial effects on change in cognition [36].

Joint effects were assessed to give insight into the mechanisms by which cognitive ability and education affects dementia risk. Additive interactions were examined along with multiplicative interactions because the former arguably has a larger public health relevance and can indicate whether there are people for whom the outcome would occur if both exposures were present but not if only one of the exposures were present [37]. Results from the analysis of additive interactions were associated with large uncertainties, and although we found positive estimates of a relative excess risk due to interaction between cognitive ability and education, all tests failed to statistically depart from their null hypothesis. Confidence intervals were especially wide for men in the high educational group with a relatively low cognitive ability because this was a rare combination in the data. Men with a relatively high cognitive ability but in the low education group were more common. We found no statistically significant evidence of interaction on either a multiplicative or additive scale (although these would be difficult to distinguish with moderate effect sizes) [38, 39]. Future studies with an increased incidence rate of dementia or total sample size are needed to establish if there could be a modifying relationship between cognitive ability and education on risk of dementia.

If the associations observed in this study are causal, the results indicate that an increase in educational attainment among the lower educated may have a protective effect on the risk of getting dementia even if it does not lead to increases in cognitive ability. Changes in compulsory schooling laws have been shown to have beneficial effects on different health outcomes including cardiovascular risk factors known to be predictive of dementia [40] and although effect sizes of education seem to be small, a reduction in risk of dementia accomplished by increasing educational attainment may be considerable because the prevalence of low education is relatively high [41]. We are also likely to have shown conservative estimates of the association between education and dementia when accounting for cognitive ability. This is because most of the men in the sample with a low or a medium education will have reached their highest educational level before completing the cognitive ability tests at the conscript board examination and the measure of cognitive ability may therefore partially mediate an effect of education on risk of dementia [16]. An earlier measure of cognitive ability would have been ideal to give a clear temporal relationship between cognitive ability and education.

Other limitations of the study include only following men for an average of 7.7 years from age 60, with a maximum age of 77, which implies that results are not necessarily generalizable to men 78 years of age or above. Nor are results generalizable to women. Relatively more women than men from the birth cohorts included in this study will be exposed to the risk of having a low education, but whether the effect of education on dementia differs for men and women is not well established [42]. Dementia cases were mainly retrieved from patient registers where the validity of a dementia diagnosis has been found to be high [26], but it is likely that there are undetected dementia cases in the patient registers. While we have no estimates of the prevalence of undetected dementia cases in a Danish context a meta-analysis estimated a European meta-rate of undetected dementia of 53.7% based on 11 studies [43]. We included dementia information from the prescription drug register to limit the number of undetected dementia cases, however, considering the small number of extra dementia cases identified using the ATC codes (8%), the number can still be expected to be substantial. It has been suggested that low education increases the risk that dementia is undiagnosed [44, 45] in which case we might expect results from this study to be a conservative estimate of the associations. We did not consider different types of dementia because of the large proportion of cases from the patient registers with an unspecified dementia diagnosis (58%), and because the validity of the individual diagnosis has been found to be low [26]. However, this is important to consider in future research with better data on dementia subtypes as associations may differ depending on the etiology of dementia. Earlier studies have, for example, suggested that the association between early life cognitive ability and dementia is stronger for vascular dementia compared to Alzheimer’s [6, 8]. Potential unadjusted confounders of the associations examined include genetics and childhood environment. Including measures of these potential confounders may attenuate the observed correlations between cognitive ability, education and risk of dementia. Another potential confounder is early psychiatric diseases. It is likely that we have very few men with early psychiatric diseases in our sample because many of these men would have been exempted from the conscript board examination [18]. The main strength of the study is the availability of high quality data on both early life measures of cognitive ability and highest attained education for a large population sample of men in risk of dementia, which allowed us to test whether education was associated with dementia independent of cognitive ability.

## Conclusions

We found both lower levels of cognitive ability in adolescence and lower educational attainment to be risk factors for dementia before age 78 among men when studied simultaneously. Results suggested that men with low educational levels and relatively low cognitive ability are a high-risk subgroup for dementia, however, the increased risk associated with exposure to both risk factors did not significantly depart from the sum of risk experienced by men only exposed to one of the risk factors alone. In other words, increases in educational attainment may at least partially offset dementia risk due to low adolescent cognitive ability.
